# Gaza: not to act is to act

**DOI:** 10.3389/ti.2026.16534

**Published:** 2026-07-31

**Authors:** Nizam Mamode, Susanna Fernandez-Diaz, Rola Tubail, Khaled Masoud, Walid Masoud

**Affiliations:** 1 Department of Renal Transplantation, Queen Elizabeth University Hospital, Glasgow, United Kingdom; 2 Department of Trauma Surgery, King Abdulaziz Hospital, Jeddah, Saudi Arabia; 3 Department of Medicine, Nasser Medical Centre, Khan Younis, Palestine; 4 Department of Vascular and Transplant Surgery, The Specialty Hospital, Amman, Jordan

**Keywords:** conflicts, gaza, hemodialysis, renal failure, transplantation

Many will be surprised to hear that prior to the start of the latest war in Gaza, on October 7th^,^ 2023, over 100 living donor renal transplants had been performed in the territory, with the hope that the programme could be expanded to match that of the West Bank, where over 500 transplants have taken place [1]. Although transplantation continues in the West Bank, in Gaza not only has transplantation stopped but patients with renal failure have no reliable treatment options. In December of last year, Nasser Hospital in Khan Younis, which is currently the main hospital within Gaza, had no peritoneal dialysis options, due to a lack of catheters, dialysis fluid and a reliable sterile environment, and had 31 functioning haemodialysis machines, with 238 dialysis patients. A four-storey dialysis centre within the hospital grounds, built just before the war, remains burnt out and unused following an attack on the hospital in June 2024. Al Aqsa hospital, the other significant hospital in the area has 23 dialysis machines, whilst a Belgian Field Hospital has 18. Three of six Government-run dialysis centres were destroyed or inactive by November 2024, with only 629 end-stage renal failure patients alive and receiving dialysis, compared with 1100 at the start of the war [2]. At that time, half the respondents in one study received 2 or fewer dialysis sessions per week, and 42% had an average of 9 days between sessions [2]. The pre-war population of Gaza was 2.4 million, with over 80% of the population displaced, resulting in over 1 million living in the central area served by Nasser Hospital; clearly the provision of care for end stage renal disease is woefully inadequate. Furthermore, access to Nasser Hospital has remained difficult-few vehicles are functioning, and prior to the recent ‘ceasefire’ movement was particularly risky due to the continued bombing. Additionally, medication for those with renal failure or a previous transplant has been difficult or impossible to obtain due to the restrictions on medical supplies. Finally, with over 1.3 million people [3] (more than half the population) living in tents or makeshift shelters lacking electricity and basic sanitation, storage of medication or fluids and adequate care of permanent lines is essentially impossible. With 71,548 fatalities in Gaza (including 20179 children) [4], directly due to trauma, it is clear that there are many further indirect deaths from the consequences of inadequate access to healthcare [5]. A Lancet publication estimated that by June 2024, when the known fatality total was 37,396, the total number of deaths likely exceeded 186,000, or 8% of the total population [6].

An unfortunate, and unprecedented, aspect of this war has been the systematic targeting of healthcare facilities. There have been 687 attacks on healthcare facilities, and 211 on ambulances and other healthcare transport, with 985 healthcare workers killed, and 306 healthcare workers detained [4]. Indeed, a quarter of Gaza’s most experienced specialists had been killed or detained by October 2024, and according to Healthcare Workers Watch ‘In Al-Shifa Hospital alone … the heads of the internal medicine department, the obstetrics and gynaecology department, the emergency department, the pathology department, the radiology department and the orthopaedic department (were killed)’. Adding to this, during the invasion of the hospital, they detained the general director, the acting head of the emergency department, the two acting heads of the orthopaedics department and the head of the anaesthesia and ICU departments.’ [7] At least three detained doctors have been tortured to death [7]. Only 18 of the pre-existing 36 hospitals are functioning, but none of these are able to provide even a basic standard of care, with constant shortages of equipment, medication and personnel. Post-operative mortality remains very high due to infection and lack of ICU care. There is currently no provision for any specialist services in the north of Gaza [4]. Hospitals which do remain open often rely on junior doctors and medical students to staff highly specialised services, such as ICU or anaesthesia. There are only two vascular surgeons working in the whole of Gaza. Prior to the war, there were 5 pathologists in Gaza, but two retired and two were killed, leaving only one pathologist to serve over two million people [7].

Despite the ceasefire, the health of most Palestinians in Gaza remains precarious. 171,353 people have been injured [4], and most of these are polytrauma, burns and blast injuries [8], which require specialist care. Famine has been an ongoing issue despite the ‘ceasefire,’ with 113 children dying from malnutrition in 2025, and a further 63,000 enrolled in treatment for malnutrition [4]. Safe elective surgery is essentially impossible due to a lack of experienced staff, basic anti-sepsis measures, lack of medication and diagnostic facilities and the poor nutritional state of the patients.

The psychological trauma of living under constant bombardment, repeated displacement, and the loss of so many friends and family members cannot be overestimated [9], and currently there are almost no services to deal with this. The use of drones adds to the trauma-prior to 2023, surveillance drones were in constant use. During the war, drones have been used to deliver explosives and other munitions, and thus represent a risk of death. Their continued presence during the “ceasefire” will not help psychological recovery.

In summary, the civilian population of Gaza have been subjected to an unprecedented, severe and collective attack for 2 years. Palestinians have not been allowed to enter or leave Gaza (only 3,140 medical evacuations have taken place [4]), and huge numbers have been killed and injured. Despite a “ceasefire,” during which 463 Palestinians have been killed [10], medical supplies and food are woefully inadequate. The healthcare system is in unable to function to a reasonable standard of secondary care, and malnutrition remains a significant problem. Some have argued that there is a distinction between medical neutrality and silence, and called on medical societies to speak out [11]. What is certainly clear is that as healthcare professionals we have a duty to consider how to help those who are in desperate need of medical care, but to whom it is denied.

## Data Availability

Publicly available datasets were analyzed in this study. This data can be found here: No datasets used.
